# SNAP and Cardiometabolic Risk in Youth

**DOI:** 10.3390/nu14132756

**Published:** 2022-07-02

**Authors:** Katelin M. Alfaro-Hudak, Lisa Schulkind, Elizabeth F. Racine, Arthur Zillante

**Affiliations:** 1 Department of Public Policy, University of North Carolina at Charlotte, Charlotte, NC 28262, USA; 2 Belk College of Business, University of North Carolina at Charlotte, Charlotte, NC 28262, USA; lschulki@uncc.edu (L.S.); azillant@uncc.edu (A.Z.); 3 College of Health and Human Services, University of North Carolina at Charlotte, Charlotte, NC 28262, USA; beth.racine@ag.tamu.edu

**Keywords:** food stamps, food security, triglycerides, dyslipidemia

## Abstract

Increasing numbers of children and adolescents have unhealthy cardiometabolic risk factors and show signs of developing metabolic syndrome (MetS). Low-income populations tend to have higher levels of risk factors associated with MetS. The Supplemental Nutrition Assistance Program (SNAP) has the potential to reduce poverty and food insecurity, but little is known about how the program affects MetS. We examine the relationship between SNAP and the cardiometabolic risk factors in children and adolescents using regression discontinuity to control for unobserved differences between participants and nonparticipants. We find that SNAP-eligible youth who experience food insecurity have significantly healthier outcomes compared to food-insecure youth just over the income-eligibility threshold. Our findings suggest that SNAP may be most beneficial to the most disadvantaged households. Policy makers should consider the broad range of potential health benefits of SNAP.

## 1. Introduction

For the first time in recent U.S. history, younger generations may not live as long as their parents or grandparents [1,2]. The increases in mortality are largely due to rising rates of obesity and chronic disease, such as cancer and cardiovascular diseases [2,3]. Disparities in life expectancy are growing, particularly across income levels, and are often related to differences in health behaviors [2]. Behavioral risk factors, such as poor diet quality and low levels of physical activity, lead to metabolic and physiological changes, such as overweight and obesity, elevated blood pressure, high cholesterol, and high blood glucose [4]. The clustering of these risk factors is known as the metabolic syndrome (MetS) [5]. Abdominal obesity, dyslipidemia, elevated blood pressure, glucose intolerance, and/or insulin resistance characterize MetS. These changes significantly increase the risk of chronic diseases, such as cardiovascular disease and type 2 diabetes. MetS has mostly been associated with adults, but the research indicates that MetS starts early in childhood [6]. The prevalence of MetS increases with the degree of obesity. Thirty percent of overweight children have MetS, and nine out of ten meet at least one of the criteria for MetS [6]. Furthermore, the presence of cardiometabolic risk factors during childhood places the child at risk of premature atherosclerosis, diabetes, organ damage, and developing insulin resistance [7]. Diet is a primary determinant of cardiometabolic health [4]. There is evidence that food insecurity is also related to increased cardiometabolic risk factors [8].

The Supplemental Nutrition Assistance Program (SNAP) is the largest federal nutrition program [9]. Nearly one in five households received benefits in 2021. (There were approximately 22 million households participating in SNAP in an average month in 2021 [10] and 122 million [11] households annually in the U.S., on average, between 2016 and 2020 [12].) Approximately 50 percent of the participants are children [13]. SNAP is targeted at low-income Americans, and household income must be at or below 130 percent of the federal poverty line (FPL) to qualify [14]. (These are the federal limits, though states have the option to expand eligibility requirements, making it easier for individuals to qualify [15]. Households must also meet employment requirements and an assets test [14].) The amount of SNAP benefits that households receive falls as household income rises [16]. The program’s stated goal is to improve food security and access to a healthier diet for low-income Americans [17]. The study findings on the relationship between SNAP and diet quality are mixed [18], but there is strong evidence that the program is associated with improved food security [19,20]. There is also evidence that increased food security is associated with a reduction in MetS risk factors among adolescents [21]. We expect that SNAP has the potential to protect against poor cardiometabolic health in low-income youth by influencing diet quality and improving food security. In addition, there is evidence that girls living in food-insecure households that participate in SNAP are less likely to be overweight than those who do not participate in SNAP, while there is no relationship between SNAP participation and overweight for those living in food-secure households [22]. For this reason, we expect the relationship between SNAP eligibility and MetS to differ by food security.

Two prior studies examined the connection between SNAP participation and cardiometabolic risk factors in youth. Neither study found a significant relationship when assessing individual risk factors [23,24]. However, when analyzing a composite score for overall cardiometabolic risk, Leung et al. [24] found that SNAP participants had a significantly higher risk score. However, it is difficult to determine whether SNAP participation or an unobserved factor is driving this association; many of the same factors that influence a child’s cardiometabolic health also affect a family’s eligibility for SNAP and their decision to participate. Generally, self-selection into the program leads to biased estimates when assessing the association between SNAP and child cardiometabolic health. No identified studies on SNAP and youth cardiometabolic health to date have used designs to control for this resulting selection bias. Understanding SNAP’s relationship with cardiometabolic health has important implications for policy and for the health of the children that SNAP serves.

To help inform this issue, we examine cardiometabolic risk factors among SNAP-eligible youth, compared to low-income youth just over the income-eligibility threshold. We apply a regression discontinuity design [25] to help account for selection bias and assess the potential role that food-security status may have in the relationship between SNAP participation and youth cardiometabolic health.

## 2. Materials and Methods

### 2.1. Data Source and Study Sample

We utilized the National Health and Nutrition Examination Survey (NHANES), an ongoing nationally representative dataset. A complete description of the data collection procedures is available elsewhere [26]. Important to note for the present analysis are the exam and laboratory components of the survey, which are conducted by trained medical professionals in a mobile examination center. Researcher-measured health indicators are substantially more accurate than self-reported or parent-reported indicators [27,28,29,30]. We use data from the 2005–2014 waves. The study population includes SNAP-eligible children and adolescents between the ages of 2 and 18; we include youth who participated in the exam and laboratory components. Demographic data and waist circumference are available for all youth. However, the data collection differs slightly across age ranges and survey components (Table 1). The analysis includes the entire age range for which each outcome measure is available.

#### 2.1.1. Measures of Cardiometabolic Health

The International Diabetes Federation (IDF) issued a consensus report in 2007, which defines the criteria for diagnosing MetS in children and adolescents [31]. This definition is the most widely used [7]. The IDF includes abdominal obesity as a prerequisite for MetS (the IDF defines abdominal obesity as having a waist circumference ≥ the 90th percentile [31]) and then defines MetS as the presence of two or more of the following factors: elevated triglycerides, low HDL cholesterol, high blood pressure, and elevated plasma glucose. We use the IDF criteria to identify our outcome risk factors and their cutoff points. (The IDF proposes that the metabolic syndrome (MetS) should not be diagnosed in children between the ages of 6 and 10 due to lack of data to identify cutoffs. However, they do suggest that children meeting the waist circumference criteria be closely monitored [31]. The IDF definition has minor differences between age ranges. For example, the cutoff for low HDL cholesterol is <40 mg/dL for both boys and girls between the ages of 10 and 16. After age 16, the cutoff for girls changes to <50 mg/dL but remains <40 for boys. However, following Leung et al. [24], we used the criteria identified in Table 1.) We examine these five risk factors both as continuous measures and as an indicator of whether the youth met the IDF criteria for the given risk factor. In addition, we use an indicator for whether the child met the criteria of having the metabolic syndrome, according to the IDF definition (Table 1). Finally, we examine a summary measure (Z-score) of overall cardiometabolic risk. Higher numbers in the cardiometabolic risk Z-score indicate higher overall cardiometabolic risk. (To create the summary measure of cardiometabolic risk, we converted each individual risk factor to Z-scores within the analytic sample and summed them to create an overall cardiometabolic risk Z-score. Of note, HDL cholesterol is a protective factor against cardiometabolic disease, where higher numbers indicate better health. To use this in the summary Z-score, we multiplied HDL by negative one and then used this to create the HDL Z-score and then the overall cardiometabolic risk Z-score. We include youth between the ages of 2 and 18 in our analysis. NHANES does not collect data on all of our outcomes of interest for all children in this age range (Table 1). We examined each individual risk factor over the entire range of ages for which it was available. The two summary measures, the one to indicate that a youth meets criteria for the metabolic syndrome and the one which is the summary Z-score, are composites of all the risk factors and thus only include youth aged 12 and older).

#### 2.1.2. Youth Food Security

Food security has been associated with worse cardiometabolic health outcomes [8] and may alter the relationship between SNAP participation and diet-related health [22]. Therefore, we stratify the analysis by food-security level. NHANES collects data on food security using questions from the U.S. Food Security Survey Module (FSSM) [32]. Eight questions pertaining specifically to youth ages 18 and younger were used to create categorical variables of child food security: (1) high food security; (2) marginal food security; (3) low food security; and (4) very low food security. We stratify our sample of youth by these four categories. It is important to note that the FSSM measures food security at the household rather than individual level. Thus, our child-level food security measure refers to the food-security status of all the children (under the age of 18) in the household.

### 2.2. Empirical Methodology

The estimation approach for this paper takes advantage of the federal income-eligibility criteria to identify how MetS factors differ in SNAP-eligible youth compared to those just over the eligibility cutoff. Using the income-eligibility criteria in a regression discontinuity design helps us to correct for the selection problem associated with studying SNAP’s relationship with child health. NHANES reports income in terms of a poverty-to-income ratio (PIR) and having a PIR equal to 1.30 is analogous to a household income equal to 130% of the FPL. We use PIR as the assignment variable. The income-eligibility criteria serve as a cutoff to distinguish the SNAP-eligible youth (household income at or below 130% of the FPL) from the ineligible (household income above 130% of the FPL). The 130% cutoff in the design mimics random assignment [33,34,35,36]. It is nearly a matter of chance if household income is just under or just above 130%.

The framework of the regression discontinuity to estimate the impact of SNAP can be understood as:

*y_i_* = *α_1_* + τ*T_i_* + *f_1_*(*X_i_* − *c*) + *T_i_*[*f_r_*(*X_i_* − *c*) − *f_1_*(*X_i_* − *c*)] + *u_i_*, (1)

where *y_i_* is the child’s health outcome; *T_i_* indicates whether or not the child lives in a SNAP-eligible household, i.e., below 130% of the FPL; *X_i_* is the household’s PIR for child *i*; *c* is the SNAP-eligibility cutoff (equal to 1.30 PIR); and *u_i_* is the error term. τ represents the intent-to-treat estimate of the SNAP program. The term (*X − c*) takes into account the distance between the cutoff and the household’s PIR for child *i*. The term *f*_1_(*X_i_ − c*) is the functional form for those with a PIR equal to or less than 1.30, who are thus eligible for SNAP; *f_r_*(*X_i_ − c*) is the functional form for those who are living above this ratio and are ineligible. This equation allows for a different relationship with the health outcome for households on either side of the cutoff. The term *T_i_*[*f_r_*(*X* − *c*) − *f_1_*(*X − c*)] is the interaction term that allows for this.

We use linear regression and linear probability models to estimate the relationship between SNAP participation and youth cardiometabolic outcomes. Youth living in households just under or just over the 1.30 PIR cutoff are expected to be similar on observable and unobservable characteristics. Therefore, our main analysis uses Equation 1 and allows for a different relationship between SNAP eligibility and the MetS outcome of interest but does not control for observable characteristics. The comparison group includes all children living in families with household income above 130% of the FPL and equal to or below 185%. Exam or fasting survey weights were used to account for the complex survey design and response rates. All analysis was conducted using Stata 14.0 (StataCorp LP: College Station, TX, USA). [37].

## 3. Results

In our sample of low-income youth, nearly 5% meet the criteria of MetS; that is, they have elevated waist circumference and two or more of the other factors (elevated triglycerides, low HDL-cholesterol, high blood pressure, and elevated plasma glucose) (Table 1). Recent prevalence estimates of MetS in children and adolescents range from 3 to 11 percent, depending on the sample and criteria used to diagnose MetS [7]. There is a comparatively high prevalence of unhealthy waist circumference, HDL cholesterol, and glucose; these are the factors that appear to be driving a diagnosis of MetS in our sample. Nearly 14% have an elevated waist circumference that is at or above the age- and sex-specific 90th percentile. HDL cholesterol—the “good” type of cholesterol—is below the IDF-specified healthy levels in 31% of our sample. Approximately, 22% of youth have elevated glucose.

**Figure 1:**
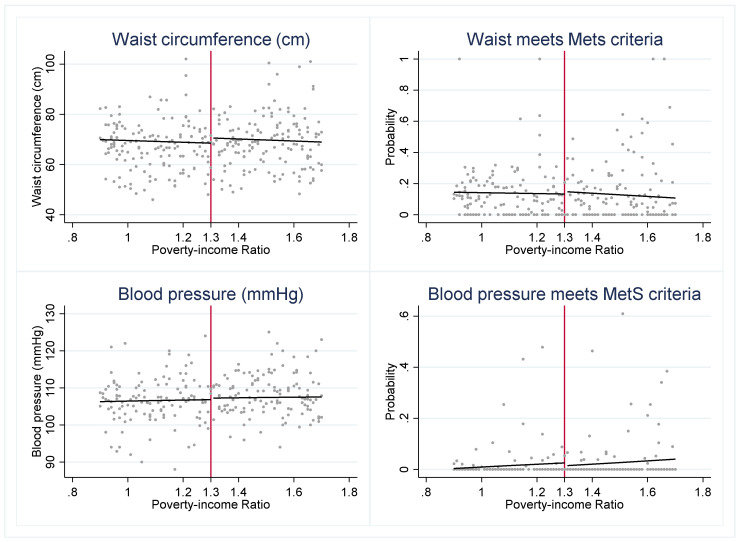

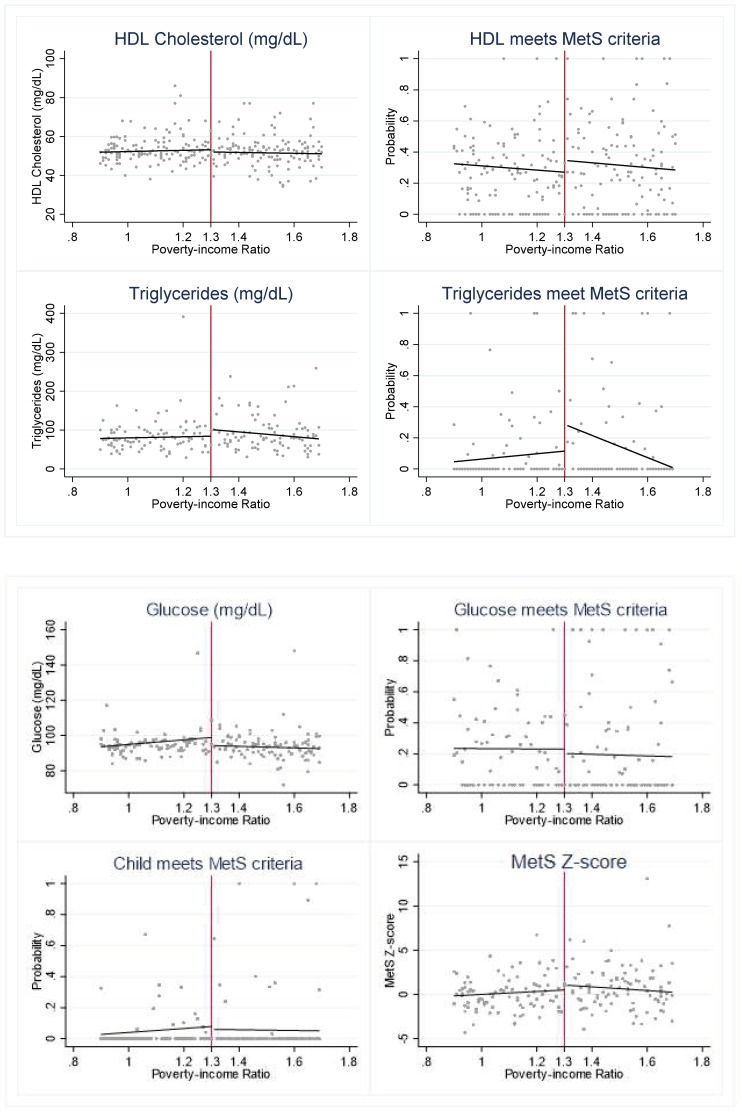
Cardiometabolic risk indicators around 1.30 poverty income ratio (PIR). Notes: Figure 1 presents the weighted, mean outcome of each cardiometabolic risk indicator across levels of PIR. The red vertical line indicates the federal SNAP income-eligibility criteria of gross household income at or below 1.30 PIR.

To investigate whether SNAP may be a protective factor for eligible youth, we first visually assess whether there is a discontinuity in the cardiometabolic risk factors across the 1.30 PIR threshold (Figure 1). We find evidence of a “jump” in several outcomes. The graphs suggest that SNAP-eligible youth have a significantly lower probability of meeting the MetS criteria for HDL and triglycerides but higher glucose levels when compared to youth just above the SNAP eligibility threshold.

The formal estimation of the relationship between SNAP eligibility and the MetS risk factors supports several of the results of the graphical analysis. In the pooled sample, we find that SNAP eligibility is associated with significantly lower triglycerides (*p* < 0.05) (Table A1), a lower probability of having elevated triglycerides (*p* < 0.05), and a lower overall MetS Z-score (*p* < 0.05) (Table 2). Furthermore, the effect of SNAP eligibility on cardiometabolic risk factors in youth varies by food-security status. We find that the significant relationship between MetS Z-score and SNAP eligibility is driven by the subsample of food-secure youth (Table 2). In addition, SNAP-eligible youth who experience either low or very low food security have significantly lower waist circumference (*p* < 0.05) (Table A1). While these results indicate that the eligibility for SNAP benefits is associated with significantly healthier cardiometabolic risk factors, we find the opposite effect in youth who are marginally food-secure. Marginally food-secure youth have significantly lower HDL (*p* < 0.05) (Table A1) and a significantly higher probability of meeting MetS criteria for HDL (*p* < 0.05) (Table 2). (HDL cholesterol is a protective factor against cardiometabolic disease, where higher numbers indicate better health. Thus, the findings in Table 2 and Table A1 are consistent in that lower HDL values (Table A1) are indicative of a less healthy cardiometabolic profile, in the same way that a higher probability of meeting the IDF’s HDL criterion (Table 2) is indicative of a less healthy cardiometabolic profile.) We test different functional forms using the pooled sample (Table A2). Our results for triglycerides and MetS Z-scores hold across multiple specifications.

If SNAP-eligible youth have several healthier MetS indicators across the levels of food security, but those who are marginally food-secure do not, what is it about marginal food security that might contribute to this difference? We examined demographic characteristics by food-security level and used multinomial logistic regression to identify significant predictors of marginal food security. (The results are not shown but are available upon request.) We find that being Black, receiving free or reduced-price school breakfast, living with a single parent or in a larger household, and household WIC participation increases the relative log odds of being marginally food-secure versus fully food-secure. Living in a household with a higher PIR increases the odds of marginal food security versus very low food security. Even so, it is unclear why marginally food-secure youth may have lower HDL levels.

### Potential Identification Issues

**Figure 2:**
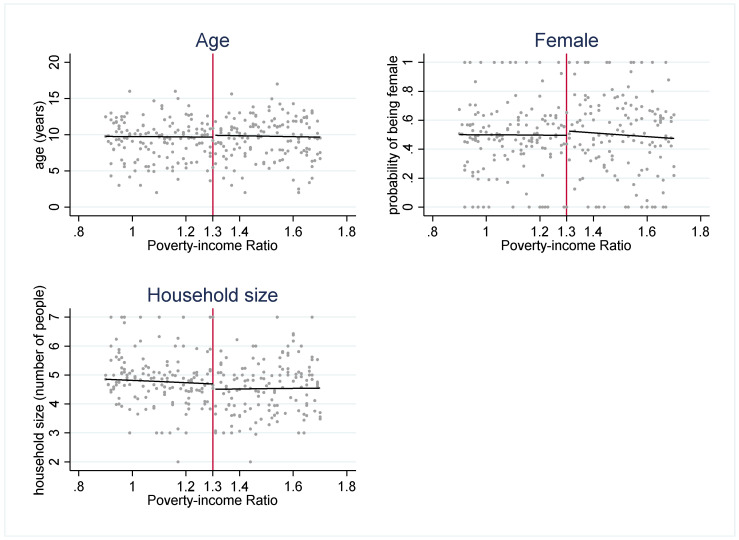
Observable characteristics around 1.30 poverty income ratio (PIR). Notes: Figure 2 plots the mean observable characteristics (age, gender, and household size) at each level of PIR. These graphs suggest that there are no systematic differences in multiple observable characteristics across the 1.30 PIR cutoff.

**Figure 3:**
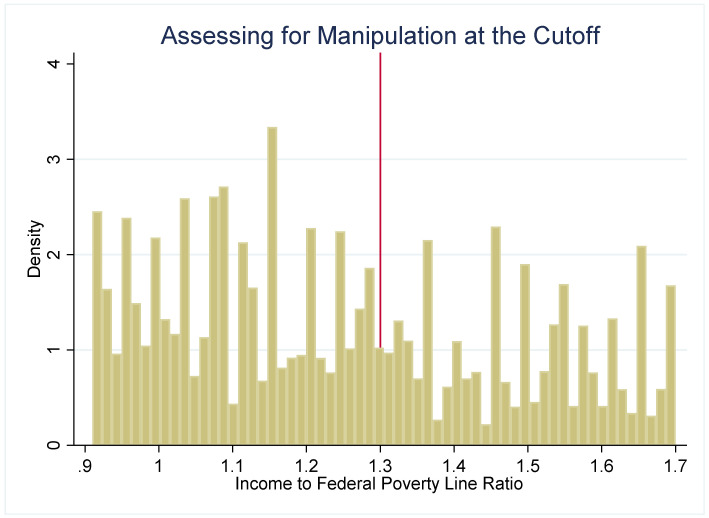
Assessing for Manipulation at the Cutoff. Notes: Figure 3 plots the density of cases across PIR to assess for manipulation at the 1.3 eligibility criteria. This figure suggests that there is a greater number of cases just over the 1.3 cutoff. We more closely examine this in Figure 4.

**Figure 4:**
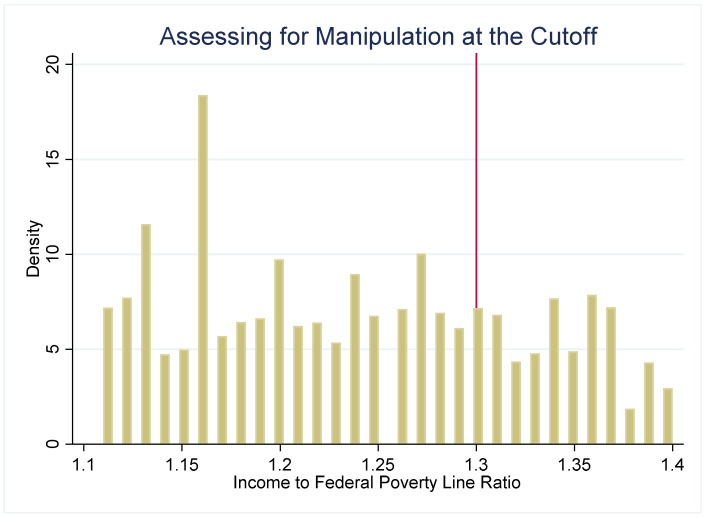
A Closer Look: Assessing for Manipulation at the Cutoff. Notes: Figure 4 plots the density of cases across PIR in a closer examination. This figure suggests that the pattern observed in Figure 3 is a result of a broader pattern, rather than manipulation of income around the cutoff.

The strength of the regression discontinuity design is that by examining outcomes in youth who are under or just over the income-eligibility criteria, the assignment to the “treatment” (SNAP eligibility) or comparison group (youth just over the threshold) becomes nearly random. To assess the plausibility of this assumption, we use several methods to check for systematic differences between the treatment and comparison groups. First, we examine how several sociodemographic characteristics (age, gender, and household size) are distributed around the 1.30 PIR threshold (Figure 2). These characteristics are distributed continuously around the threshold, which suggests that youth just under and just over the 1.30 cutoff have similar observable characteristics. Second, if either SNAP administrators or potential SNAP participants can precisely manipulate household income, then the assignment to treatment or control is no longer nearly random. We plot the density of PIR to assess if income manipulation may be occurring. If this were the case, then it would be very likely that households just under and just over the cutoff differ systematically on unobservable characteristics. Figure 3 and Figure 4 do not suggest that income manipulation is occurring.

Next, we examine the sensitivity of the regression results to the choice of bandwidth, i.e., the choice of what levels of PIR to include in specifying who is in the treatment and comparison groups. The main analysis includes all youth who live in households with an income at or below 1.85 PIR. The treatment group includes youth with a household income at or below 1.30 PIR. The comparison group are youth with a household income above 1.30 but at or below 1.85 PIR. Table A3 presents the results using the same comparison group but a narrower treatment group. Here, the treatment group includes youth with a household income above 0.80 PIR but equal to or below 1.30 PIR. The results are generally robust with regard to the choice of bandwidth, with a few exceptions. As in the main analysis, SNAP-eligible youth have significantly lower triglycerides and MetS Z-scores. Interestingly, we also find that when using a narrower treatment group the SNAP-eligible youth have significantly *higher* glucose than those just over the eligibility threshold. These regression results corroborate the graphical analysis of the relationship between SNAP eligibility and glucose.

**Figure A1:**
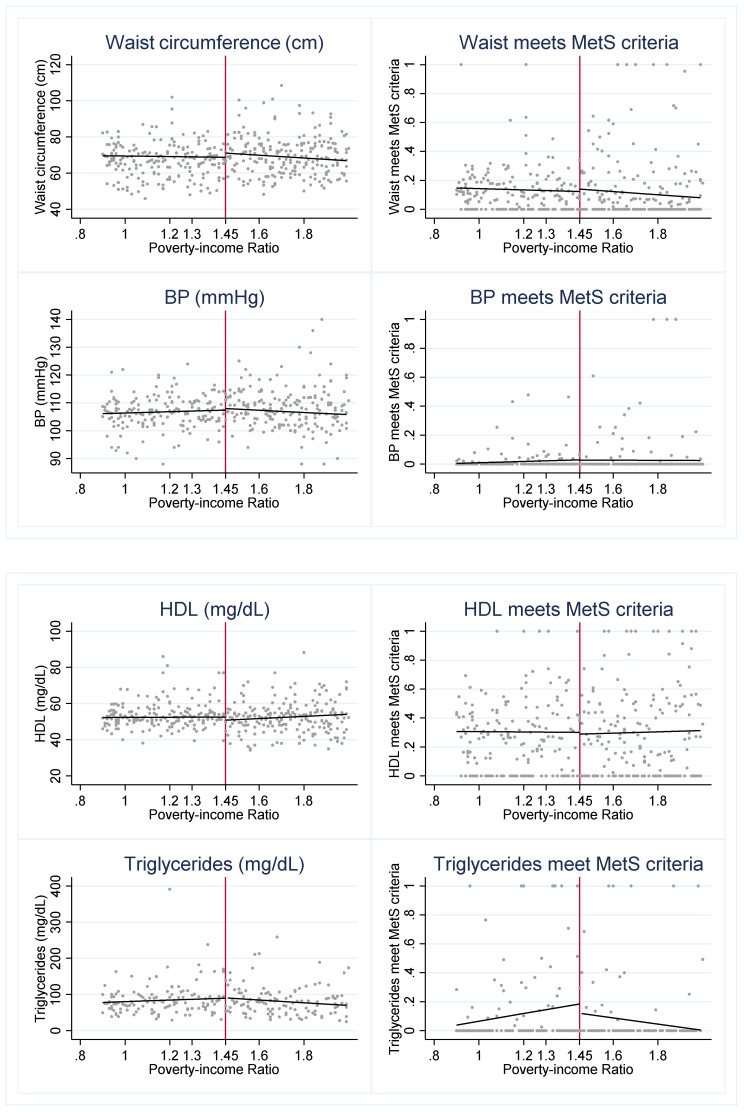

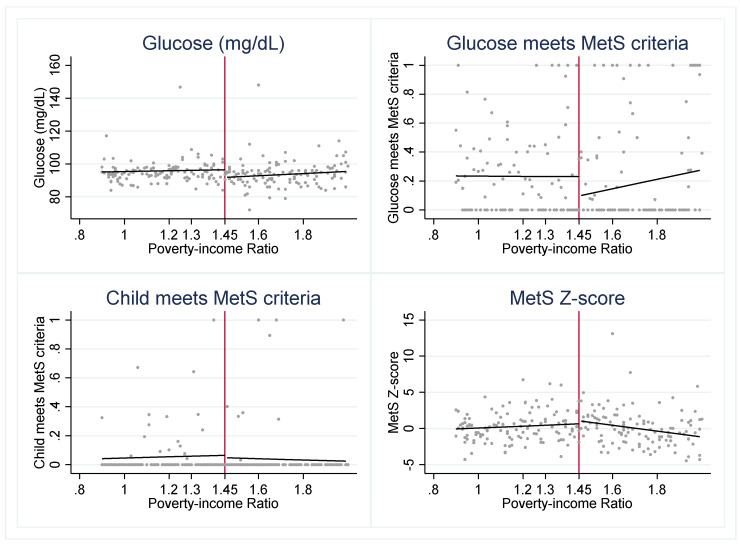
Outcomes across 1.45 PIR Notes: Figure A1 presents the weighted, mean outcome of each cardiometabolic risk indicator across levels of PIR. The red vertical line indicates 1.45 PIR.

Finally, if the intent-to-treat estimate of SNAP was the reason for our findings, then we would not expect to see a similar discontinuity in outcomes across the different levels of PIR. To evaluate this question, we visually check for a jump in outcomes across 1.45 PIR (Figure A1), which, to the best of our knowledge, is not used as an income cutoff for any program. The graphs suggest that there is a discontinuity in the two outcomes. There is evidence that those just under 1.45 PIR have a higher probability of meeting MetS criteria for high triglycerides and for high glucose. When using 1.30 PIR, the graphs suggest that SNAP-eligible youth have a *lower* probability of meeting the MetS criteria for triglycerides. The regression results provide evidence that this relationship is significant. As we find the opposite association in the falsification test, we are more confident that significantly lower triglycerides in SNAP-eligible youth are not driven by an outside factor. We formally tested for whether 1.45 PIR was significantly associated with MetS indicators using the same framework as our main analysis. Although we generally find null results when testing the impact of a fictitious program with a 1.45 PIR cutoff, there is evidence that several outcomes are significantly associated with a 1.45 PIR cutoff (Table A4). These findings may highlight a weakness in using an RD when studying SNAP. That is, although having gross household income equal to or below 1.30 PIR is the basic federal eligibility criteria, eligible deductions (e.g., childcare costs and excess shelter costs) may make households with income slightly over the 1.30 cutoff actually eligible for SNAP. Our findings must be viewed with this caveat in mind.

## 4. Discussion

This study uses a quasi-natural experiment to investigate the relationship between SNAP and the cardiometabolic health of children and adolescents. We find that SNAP-eligible youth have significantly healthier outcomes (lower triglycerides, lower probability of triglycerides meeting MetS criteria, and lower overall MetS Z-scores) compared to youth just over the eligibility threshold. In addition, the connection between SNAP and health outcomes varies by a youth’s food-security status. Our analysis indicates that SNAP eligibility is associated with *healthier* outcomes in youth who are fully food-secure or who experience low or very low food security.

Consistent with Jones et al., we find that the relationship between SNAP participation and diet-related health differs by food-security status, though we examine four categories of food security at the youth level, while Jones et al. compared two categories which were at the household level [30]. We find that SNAP eligibility is associated with a significantly healthier MetS Z-score in youth who are fully food-secure and with waist circumference in youth who experience low or very low food security. However, we find that marginally food-secure youth have significantly lower, and therefore less healthy, HDL levels and a higher probability of having MetS. Similarly, Parker et al. found that adults with marginal or very low food security had increased odds of having MetS [8]. Likewise, Tester et al. found that marginally food-secure adolescents had increased odds of elevated triglycerides and a less healthy blood lipid profile compared to fully food-secure adolescents [21]. They did not find evidence of unhealthy lipid levels in youth with low or very low food security.

It is important to note that we evaluate and present the intent-to-treat estimate of SNAP. That is, we estimate cardiometabolic indicators for all income-eligible youth, compared to those just over the income-eligibility threshold. We take this approach to mitigate the issue of selection bias. Households who choose to apply and participate in SNAP differ in both observable and unobservable characteristics from income-eligible households who choose not to participate in SNAP. This resulting selection bias creates challenges in accurately estimating the relationship between SNAP participation and health outcomes. Bitler examines the differences between SNAP participants and income-eligible nonparticipants and finds that, among other differences, SNAP participants have lower quality diets, are more likely to be obese, and have lower overall health [38]. Importantly, Bitler notes that many of these differences likely predate participation in SNAP, illustrating that selection into SNAP complicates any evaluation of the program [38]. Similarly, Leung et al. found that the adolescent SNAP participants had lower overall diet quality than the income-eligible nonparticipants [24]. The study authors also investigated demographic differences and found that, compared to the income-eligible nonparticipants, the SNAP participants were more likely to be a racial/ethnic minority, live in a single-parent home, have a larger household size, and have a parent with fewer years of education [24]. In addition, SNAP participation is underreported by an estimated 11 to 50% [39,40], further exacerbating the problem of bias. Therefore, we conduct an intent-to-treat analysis rather than comparing self-reported SNAP participants to nonparticipants.

### 4.1. Limitations

The present study has several strengths. To our knowledge, this is the first study to use a quasi-experimental design to investigate the relationship between SNAP and cardiometabolic risk factors in children and adolescents. In addition, we examine whether food-security status modifies the link between SNAP and youth risk factors. This paper also has limitations. Even though we use a design that addresses selection bias and attempts to control for both observable and unobservable characteristics, the data we use are observational. For that reason, we are limited in our ability to draw causal inferences. Secondly, due to complex institutional rules surrounding SNAP eligibility, it is likely that we will misclassify some youth as being in the SNAP-eligible group or in the nearly eligible group. Finally, Hoynes and Schanzenbach note that RD may not be suitable for analyzing SNAP because the amount of benefits received falls as income rises. Therefore, the discontinuity between receiving benefits and not receiving any benefits is not as sharp a discontinuity as that with programs such as the National School Lunch Program [41]. However, the American Recovery and Reinvestment Act of 2009 (ARRA) was in effect during most of the years of our study data. The ARRA increased SNAP benefit levels for all participants. Beginning in April 2009, SNAP benefits increased by an average of 19 percent [42]. The benefits increased by a constant dollar amount for each household size regardless of income [43]. The difference in benefits received even in SNAP households that were close to the cutoff was therefore more pronounced between 2009 and 2013, during the period of the ARRA.

### 4.2. Policy Implications and Future Research

Given the prevalence of cardiometabolic risk factors in increasing numbers of youth, it is essential to understand the role that the largest U.S. nutrition assistance program may play. However, the selection effect creates special challenges in elucidating the connection between youth cardiometabolic health and SNAP. Multiple researchers have called for ethical, random-assignment designs in order to clarify the relationship between SNAP and participant health [44,45]. No recent studies have been successful in implementing such a design. Even so, several groups of researchers have conducted projects worth noting here. Mathematica Policy Research conducted the SNAP Food Security (SNAPFS) survey for the USDA Food and Nutrition Service in 2011 and 2012 [19]. The SNAPFS survey examined the differences in food-security levels between households that had been accepted into SNAP but were not yet receiving benefits and households that had been receiving benefits for several months. The study found that SNAP participation was associated with improved food security at both the child and the household levels [19]. Studies such as the USDA Healthy Incentives Pilot [11] and smaller, independent researcher-funded studies [46] have assessed how purchase incentives and restrictions may affect purchases. Designs such as those used in these studies could be extended to study the connection between SNAP and child health. Legislators may consider authorizing projects that use random-assignment or quasi-experimental research designs in order to clarify the impact of SNAP on participant health.

Secondly, we find that SNAP may be most beneficial for children and adolescents who experience either low or very low food security. If additional research corroborates these results, policy makers may examine the possibility of targeting additional SNAP benefits to segments of the population that are most likely to experience food insecurity. This question—as well as our finding that SNAP may be particularly salubrious for food-insecure youth—becomes more salient in light of two recent policy changes: (1) the 15% increase in SNAP benefits that was in response to the COVID-19 pandemic expired in September 2021 and (2) The Thrifty Food Plan (TFP) that was revised in October 2021, which effectively increased benefit amounts to provide an additional 40 cents per meal. First, the Consolidated Appropriations Act, 2021 [47] and then the American Rescue Plan Act of 2021 [48] increased the amount of SNAP benefits by 15% from 1 January 2021 through to 30 September 2021. The temporary benefit increase was designed to help families purchase food during a time when many were experiencing additional financial hardship due to the COVID-19 pandemic. Just as the COVID-19-specific benefit increase ended, the revisions to the TFP went into effect. The TFP provides the basis for SNAP benefit calculations. Although the TFP has been updated regularly since it came into use in 1975, historically the updates have been cost neutral while adjusting for inflation. However, the TFP 2021 was calculated based on a reevaluation of the existing data on the cost of a healthy and practical diet, without the requirement of cost neutrality. The result was an average increase of 21% for a family of four, which was the first time in 40 years that the real value of the TFP increased [49]. It has yet to be seen whether these benefit increases will affect food security and diet-related health markers in youth. Even so, previous research on the SNAP benefit increase that was part of the ARRA can shed light on this question. The 19% benefit increase provided a natural experiment to assess the connection between a SNAP benefit increase and food security. Two recent studies that have leveraged this benefit increase to examine the relationship between an increase in SNAP benefits and food security have had mixed results. While an analysis of the Current Population Survey found that household food insecurity decreased by 2.2 percentage points [50], an analysis of NHANES found that child-level food security was not significantly associated with the ARRA SNAP benefit increase [51]. Future research should investigate whether the two 2021 SNAP benefit increases affected food security and diet-related health, including cardiometabolic health, among youth.

As noted, lower-income families receive a higher amount of SNAP benefits than higher-income families. Therefore, SNAP’s benefit structure is already intended to provide greater resources to more financially disadvantaged households. However, although income partially explains food-security status, several other factors are associated with child food insecurity, even when controlling for income. Having a larger share of the household between the ages of 13 and 18, lacking an emotional or financial support system, and having a household head with poor physical or mental health, are all associated with child food insecurity [52]. Recent work by Nikolaus et al. found evidence that “grit”—i.e., perseverance and passion to reach a goal [53]—may be one previously overlooked factor that enables some low-income households to be food-secure. The study authors found that parents with more grit had significantly lower odds of household food insecurity. Similarly, children with more grit had significantly lower odds of child food insecurity [54]. Although some of these components may fall far beyond the scope of the program (e.g., assessing the physical and mental health of the household head), there may be feasible ways to further target benefits to those who are most likely to experience food insecurity. For example, as households with a larger proportion of adolescents are at a higher risk of child food insecurity, policy makers may consider incorporating the ages of children, instead of only the number of children in the household, into the SNAP benefit formula.

Additional research is needed to illuminate the relationship between SNAP and the cardiometabolic health of children and adolescents. First, future studies should use research designs that address selection bias. In addition, researchers should use longitudinal data in order to study the dynamics of program participation, child health, and important factors such as food security, if/when the data become available. Finally, studies should examine the causal mechanisms through which SNAP is linked with youth cardiometabolic health. The complexities of SNAP—and of the policy process—create significant challenges in studying the effects of SNAP and in making changes to the program. However, continuing research in these areas will help clarify the relationship between SNAP and cardiometabolic risk factors in children and adolescents. A greater understanding of the broader health impacts of SNAP can help inform policy discussions on program structure, funding levels, and potential program changes. Refining the largest nutrition assistance program has the potential to improve the cardiometabolic health of low-income youth.

## Figures and Tables

**Table 1:** Definitions of Abnormal Values for Risk-Factor Variables.

Risk Factor	Age Range Available in NHANES	No. of Participants Evaluated	Definition of Abnormal Value	Sample Mean (SE)	Percent Meeting Criteria
**Waist circumference (cm)**	2–18	9226	≥90th percentile	78.15 (0.40)	13.85% (0.75)
**HDL cholesterol (md/dL)**	6–18	6019	<40 mg/dL in boys <50 mg/dL in girls	51.77 (0.31)	31.35% (1.09)
**Systolic blood pressure (mmHg)**	8–18	5257	≥130 mmHg	107.19 (0.24)	2.07% (2.43)
**Triglycerides (mg/dL)**	12–18	1570	≥150 mg/dL	84.47 (1.88)	8.64% (1.01)
**Glucose (mg/dL)**	12–18	1590	≥100 mg/dL	94.48 (0.49)	21.57% (1.81)
**MetS**	12–18	1452	Elevated waist circumference and 2+ risk factors	-	4.96% (0.89)
**MetS Z-score**	12–18	1427	Sum of risk factor Z-scores	0.27 (0.10)	-

Notes: International Diabetes Federation [31] criteria for risk-factor variables and metabolic syndrome in children and adolescents. Table 1 also presents the age range for which each risk-factor is available in NHANES and the sample size used in the analysis. The summary variables used in the analysis (MetS binary and MetS Z-score) were created using all the risk-factor variables and are only available if the youth had reliable data for each specific indicator. Sample mean and standard error of the analyzed population are presented in the last column. HDL: high-density lipoprotein cholesterol; MetS: metabolic syndrome.

**Table 2:** Estimated effect of SNAP eligibility on cardiometabolic risk factors in children and adolescents compared to those just over the eligibility threshold: differences across food security.

	(Column 1)	(Column 2)	(Column 3)	(Column 4)	(Column 5)
	Pooled Sample	High	Marginal	Low	Very Low
Risk Factor	(*n* = 9614)	(*n* = 5977)	(*n* = 979)	(*n* = 1544)	(*n* = 236)
**Waist criteria**	−0.01 (0.02)	0.00 (0.02)	−0.02 (0.07)	−0.11 * (0.05)	0.02 (0.11)
**BP criteria**	−0.01 (0.01)	0.00 (0.01)	−0.04 (0.04)	−0.04 (0.04)	−0.09 (0.09)
**HDL criteria**	−0.04 (0.04)	−0.07 (0.05)	0.27 * (0.11)	0.02 (0.08)	−0.33 (0.25)
**Triglycerides criteria**	−0.12 * (0.05)	−0.08 (0.06)	−0.02 (0.07)	−0.18 (0.14)	−0.11 (0.14)
**Glucose criteria**	−0.08 (0.07)	−0.12 (0.08)	0.15 (0.19)	−0.06 (0.17)	-
**MetS Z-score**	−1.08 * (0.48)	−1.81 ** (0.59)	0.08 (1.34)	0.54 (1.65)	0.84 (1.84)

Standard errors in parentheses ** *p* < 0.01, * *p* < 0.05. Notes: Table 2 presents the estimated effect of living in a SNAP-eligible household on youth cardiometabolic risk factors (τ, from Equation (1)). Each coefficient presented is from a separate regression, with the risk factor outcome variable in the far left column. Column 1 displays the estimates for the pooled sample (i.e., all levels of food security). Columns 2 through 5 display estimates by food security level. All models are unconditional, linear fits that center PIR on the 1.30 cutoff and allow for a different relationship between SNAP eligibility and each risk factor on either side of the cutoff. The model for meeting glucose criteria among youth with very low food security is unable to be estimated due to a small n. All models account for the complex survey design. We also checked various specifications for each outcome to assess different functional forms (Table A2). We focus on a linear fit because the graphs do not suggest a quadratic or cubic relationship. Variable names are abbreviated. Variables denoted “criteria” refer to the probability of meeting IDF criteria for metabolic syndrome for that specific component. BP: blood pressure; HDL: high-density lipoprotein cholesterol; MetS: metabolic syndrome.

## Data Availability

NHANES data are publicly available from the National Center for Health Statistics at the CDC.

## Appendix A

**Table A1:** Estimated effect of SNAP eligibility on cardiometabolic risk factors in children and adolescents compared to those just over the eligibility threshold: differences across food security (additional outcomes).

	(Column 1)	(Column 2)	(Column 3)	(Column 4)	(Column 5)
	Pooled Sample	High	Marginal	Low	Very Low
Risk Factor	(*n* = 9614)	(*n* = 5977)	(*n* = 979)	(*n* = 1544)	(*n* = 236)
Waist (cm)	−1.98 (1.54)	−1.21 (1.76)	2.13 (3.67)	−6.80 * (2.75)	−14.74 * (7.15)
BP (mmHg)	−1.56 (0.96)	−1.05 (1.20)	−1.78 (3.08)	−4.57 (2.44)	−3.17 (3.95)
HDL (mg/dL)	0.98 (1.19)	1.71 (1.30)	−6.24 * (2.62)	1.83 (3.28)	4.75 (6.08)
Triglycerides (mg/dL)	−17.31 * (7.83)	−15.09 (10.54)	−20.46 (18.85)	−16.61 (16.28)	6.27 (31.08)
Glucose (mg/dL)	3.22 (1.89)	3.22 (2.46)	6.75 (5.77)	3.06 (6.42)	2.97 (2.86)
Youth meets criteria for MetS	−0.06 (0.05)	−0.11 (0.07)	0.02 (0.11)	-	-

Standard errors in parentheses * *p* < 0.05. Notes: Table A1 presents the estimated effect of living in a SNAP-eligible household on additional youth cardiometabolic risk factors (τ, from Equation (1)). Each coefficient presented is from a separate regression, with the risk factor outcome variable in the far left column. Column 1 displays the estimates for the pooled sample (i.e., all levels of food security). Columns 2 through 5 display estimates by food-security level. All models are unconditional, linear fits that center PIR on the 1.30 cutoff and allow for a different relationship between SNAP eligibility and each risk factor on either side of the cutoff. All models account for the complex survey design. We also checked various specifications for each outcome to assess different functional forms (Table A2). We focus on a linear fit because the graphs do not suggest a quadratic or cubic relationship. Variable names are abbreviated. Models 4 and 5 are unable to be estimated for their meeting of MetS criteria due to a small n. BP: blood pressure; HDL: high-density lipoprotein cholesterol; MetS: metabolic syndrome.

**Table A2:** Estimated effect of SNAP eligibility on cardiometabolic risk factors in children and adolescents compared to those just over the eligibility threshold: different functional forms. (Model 1 is an unconditional model that centers PIR on 1.30 but does not allow the slope to vary on either side of the 1.30 PIR cutoff. Model 2 is the same as Model 1 (i.e., slopes do not vary) but controls for youth-specific characteristics (age, sex, race, food-security status, and participation in nutrition assistance programs) and household-level controls (HR education, HR marital status, HH size). Models 3 through 7 also include these controls. Model 3 adds an interaction term between the SNAP treatment variable and has a centered PIR, which allows the slope to vary on either side of the PIR cutoff. Model 3 is the same model as the main model presented in the manuscript for the pooled sample (Table 2, Column 1), but adds controls. Model 4 is a quadratic model that includes PIR (centered on 1.30) and centered PIR². Model 5 is a quadratic interaction model that includes centered PIR, centered PIR², and interactions between the SNAP treatment variable and both PIR and PIR². Model 6 is a cubic model in which the slopes are identical on either side of the PIR cutoff, but it includes cubic PIR. Model 7 is a cubic interaction model that includes cubic PIR and allows the slopes to vary on either side of the cutoff. These different functional forms mirror those described by Jacob et al. [1].)

Unconditional		Includes Controls					
	(Model 1)	(Model 2)	(Model 3)	(Model 4)	(Model 5)	(Model 6)	(Model 7)
Risk Factor	(*n* = 9614)	(*n* = 8749)	(*n* = 8749)	(*n* = 8749)	(*n* = 8749)	(*n* = 8749)	(*n* = 8749)
Waist (cm)	−0.45 (1.13)	−0.08 (0.74)	−1.25 (0.90)	−1.49 (0.92)	−1.07 (1.23)	−1.48 (0.92)	−1.73 (1.74)
Waist criteria	0.02 (0.02)	0.02 (0.02)	−0.01 (0.02)	−0.01 (0.02)	−0.01 (0.03)	−0.01 (0.02)	−0.00 (0.04)
BP (mmHg)	−1.00 (0.83)	−0.34 (0.81)	−1.25 (0.89)	−1.04 (1.07)	−0.24 (1.23)	−1.02 (1.08)	0.18 (1.67)
BP criteria	−0.01 (0.01)	−0.00 (0.01)	−0.01 (0.01)	−0.00 (0.01)	−0.00 (0.01)	−0.00 (0.01)	0.00 (0.02)
HDL (mg/dL)	0.33 (1.01)	0.97 (0.94)	1.49 (1.14)	2.33 * (1.10)	1.58 (1.50)	2.35 * (1.09)	2.04 (2.10)
HDL criteria	−0.02 (0.03)	−0.04 (0.03)	−0.05 (0.04)	−0.07 + (0.04)	−0.08 (0.06)	−0.07 + (0.04)	−0.05 (0.08)
Triglycerides (mg/dL)	−5.67 (5.52)	−7.96 (5.47)	−19.36 * (7.83)	−20.17 * (8.73)	−20.23 + (11.78)	−20.37 * (8.66)	−26.01 (16.50)
Triglycerides criteria	−0.04 (0.04)	−0.05 (0.04)	−0.12 * (0.05)	−0.10 + (0.06)	−0.12 (0.08)	−0.10 + (0.06)	−0.19 + (0.11)
Glucose (mg/dL)	3.35 (2.18)	5.03 * (1.92)	3.67 * (1.65)	4.76 + (2.71)	1.06 (2.65)	4.73 + (2.65)	0.18 (3.82)
Glucose criteria	−0.01 (0.05)	0.02 (0.05)	−0.08 (0.07)	−0.10 (0.07)	−0.05 (0.10)	−0.10 (0.07)	−0.20 (0.13)
MetS criteria	−0.02 (0.03)	−0.04 (0.03)	−0.10 + (0.05)	−0.08 (0.05)	−0.16 + (0.09)	−0.08 (0.05)	−0.28 + (0.15)
MetS Z-scores	−0.40 (0.39)	−0.49 (0.42)	−1.46 ** (0.52)	−1.62 ** (0.53)	−1.02 (1.03)	−1.63 ** (0.53)	−0.84 (1.56)

** *p* < 0.01, * *p* < 0.05, + *p* < 0.1. Notes: Table A2 presents the estimated effect of living in a SNAP-eligible household on youth cardiometabolic risk factors (τ, from Equation (1)) across different functional forms. Variable names are abbreviated. Variables denoted “criteria” refer to the probability of meeting IDF criteria for metabolic syndrome for that specific component [31]. All models account for the complex sampling design of NHANES. BP: blood pressure; HDL: high-density lipoprotein cholesterol; MetS: metabolic syndrome.

**Table A3:** Estimated effect of SNAP eligibility on cardiometabolic risk factors in children and adolescents between the ages of 2 and 18 years: narrow bandwidth across functional forms.

	(Model 1)	(Model 2)	(Model 3)	(Model 4)	(Model 5)	(Model 6)	(Model 7)	(Model 8)
Risk Factor	(*n* = 5715)	(*n* = 5296)	(*n* = 5715)	(*n* = 5296)	(*n* = 236)	(*n* = 5296)	(*n* = 5296)	(*n* = 5296)
Waist (cm)	−1.26 (1.53)	−1.32 (1.06)	−1.53 (1.58)	−1.47 (1.06)	−1.44 (1.06)	−1.94 (1.37)	−1.87 (1.26)	−2.66 (1.98)
Waist criteria	−0.00 (0.02)	−0.00 (0.02)	−0.01 (0.02)	−0.01 (0.02)	−0.00 (0.02)	−0.02 (0.03)	−0.02 (0.03)	−0.02 (0.05)
BP (mmHg)	−1.03 (1.19)	−0.77 (1.18)	−1.16 (1.18)	−0.92 (1.16)	−0.85 (1.17)	0.42 (1.35)	0.16 (1.24)	0.39 (1.84)
BP criteria	0.00 (0.01)	0.00 (0.01)	−0.00 (0.01)	0.00 (0.01)	0.00 (0.01)	0.01 (0.02)	0.01 (0.02)	0.01 (0.02)
HDL (mg/dL)	1.10 (1.22)	1.65 (1.20)	1.19 (1.24)	1.71 (1.22)	1.70 (1.22)	1.38 (1.84)	1.37 (1.63)	3.01 (2.47)
HDL criteria	−0.04 (0.05)	−0.04 (0.04)	−0.04 (0.05)	−0.04 (0.04)	−0.04 (0.04)	−0.08 (0.07)	−0.07 (0.06)	−0.10 (0.09)
Triglycerides (mg/dL)	−15.95 + (8.61)	−19.58 * (8.69)	−18.21 * (8.93)	−22.20 * (8.83)	−21.08 * (8.67)	−21.27 (12.96)	−18.85 + (11.25)	−36.95 * (17.04)
Triglycerides criteria	−0.07 (0.05)	−0.08 (0.06)	−0.09 + (0.05)	−0.10 + (0.06)	−0.09 (0.06)	−0.12 (0.08)	−0.09 (0.07)	−0.25 * (0.11)
Glucose (mg/dL)	5.87 + (3.32)	5.71 * (2.67)	5.39 + (2.97)	4.99 * (2.33)	5.42 * (2.53)	3.01 (4.18)	4.60 (4.38)	3.34 (5.35)
Glucose criteria	−0.04 (0.07)	−0.04 (0.07)	−0.06 (0.07)	−0.07 (0.07)	−0.06 (0.07)	−0.06 (0.10)	−0.08 (0.09)	−0.20 (0.13)
MetS criteria	−0.03 (0.05)	−0.07 (0.05)	−0.04 (0.05)	−0.08 (0.05)	−0.08 (0.05)	−0.17 + (0.09)	−0.12 + (0.07)	−0.31 * (0.14)
MetS Z-scores	−0.88 (0.57)	−1.26 * (0.62)	−1.03 + (0.59)	−1.48 * (0.63)	−1.40 * (0.62)	−0.75 (1.07)	−0.84 (0.86)	−1.46 (1.54)

Standard errors in parentheses * *p* < 0.05, + *p* < 0.1. Notes: Table A3 presents the estimated effect of living in a SNAP-eligible household on youth cardiometabolic risk factors (τ, from Equation (1)) for youth aged 2 to 18 years or the age range for which the outcome is available. The main RD analysis includes all youth who live in households with income at or below 1.85 PIR. The treatment group includes youth with household incomes at or below 1.30 PIR. The comparison group comprises youth with household incomes above 1.30 but at or below 1.85 PIR. Table A3 presents results using the same comparison group but a narrower treatment group. Here, the treatment group includes youth with household incomes above 0.80 PIR but equal to or below 1.30 PIR. Variable names are abbreviated. Variables denoted “binary” refer to the probability of meeting IDF criteria for metabolic syndrome for that specific component. All models account for the complex sampling design of NHANES. The number of observations noted at the top of each column is for waist circumference and the probability of meeting metabolic syndrome criteria. The number of observations for other outcomes is smaller and is available upon request.

**Table A4:** Falsification test—using PIR 1.45.

	(Model 1)	(Model 2)	(Model 3)	(Model 4)	(Model 5)
	Pooled Sample	High	Marginal	Low	Very Low
Risk Factor	(*n* = 9963)	(*n* = 6261)	(*n* = 996)	(*n* = 1587)	(*n* = 236)
Waist (cm)	−2.27 (1.75)	−2.65 (2.03)	2.53 (3.26)	−1.12 (3.97)	0.25 (7.57)
Waist criteria	−0.01 (0.02)	−0.02 (0.03)	0.13 * (0.06)	−0.02 (0.08)	−0.19 (0.33)
BP (mmHg)	−1.14 (1.35)	−1.28 (1.48)	2.24 (4.21)	−2.42 (2.58)	−13.26 * (6.23)
BP criteria	−0.01 (0.01)	−0.01 (0.01)	−0.02 (0.05)	0.03 + (0.02)	-
HDL (mg/dL)	1.13 (1.40)	1.22 (1.64)	−0.02 (2.55)	1.54 (3.33)	−3.60 (11.10)
HDL criteria	0.01 (0.05)	0.03 (0.06)	0.13 (0.09)	−0.17 (0.14)	0.12 (0.31)
Triglycerides (mg/dL)	−8.00 (7.91)	−9.17 (8.37)	36.57 * (16.53)	15.38 (16.51)	-
Triglycerides criteria	−0.02 (0.05)	−0.01 (0.05)	0.20 * (0.10)	−0.02 (0.11)	-
Glucose (mg/dL)	5.02 * (1.98)	5.26 * (2.43)	15.31 ** (4.54)	6.08 (4.12)	-
Glucose criteria	0.09 (0.07)	0.02 (0.09)	0.53 ** (0.14)	0.25 ** (0.09)	0.04 (0.00)
MetS criteria	−0.01 (0.04)	−0.03 (0.05)	0.31 ** (0.04)	-	-
MetS Z-scores	−1.06 * (0.52)	−1.19 * (0.58)	2.92 ** (0.63)	−2.22 * (0.98)	5.02 ** (0.97)

Standard errors in parentheses * *p* < 0.05, + *p* < 0.1. Notes: This table presents the results of an assumption check. It presents the intent-to-treat estimate of a fictious program (τ, from Equation (1)) that has an income-eligibility cutoff of 1.45 PIR. This equation controls for whether a child_i_ lives in a household with gross income at or below 1.45 PIR, a variable equal to a child_i_’s PIR, centered on 1.45, and an interaction term between the fictitious SNAP program and the centered PIR. All estimates present a basic, unconditional linear model that allows the slope to vary on either side of the cutoff. Model 1 displays the RD estimates for the pooled sample (i.e., all levels of food security). Models 2 through 5 display estimates by food-security level. The number of observations reported refers to the sample for regressions with waist circumference as an outcome. Models include the full age range for which data are available; therefore, the number of observations differ across models. The number of observations becomes very small in some models. Variables that use a smaller, fasted sample (e.g., triglycerides, glucose, MetS Z-score) have an *n* as low as 42 among youth with very low food security. Due to a small n, some models are unable to be estimated. All models account for the complex survey design. Variable names are abbreviated. Variables denoted “criteria” refer to the probability of meeting IDF criteria for metabolic syndrome for that specific component. BP: blood pressure; HDL: high-density lipoprotein cholesterol; MetS: metabolic syndrome.
